# Dynamic transcriptomic and regulatory networks underpinning the transition from fetal primordial germ cells to spermatogonia in mice

**DOI:** 10.1111/cpr.13755

**Published:** 2024-09-27

**Authors:** Jiexiang Zhao, Kang Tang, Gurong Jiang, Xinyan Yang, Manman Cui, Cong Wan, Zhaoxiang Ouyang, Yi Zheng, Zhaoting Liu, Mei Wang, Xiao‐Yang Zhao, Gang Chang

**Affiliations:** ^1^ The Tenth Affiliated Hospital Southern Medical University (Dongguan People's Hospital) Dongguan Guangdong PR China; ^2^ State Key Laboratory of Organ Failure Research, Department of Developmental Biology, School of Basic Medical Sciences Southern Medical University Guangzhou Guangdong PR China; ^3^ School of Traditional Chinese Medicine Southern Medical University Guangzhou Guangdong PR China; ^4^ Maoming People's Hospital Maoming Guangdong PR China; ^5^ Guangdong Provincial Key Laboratory of Construction and Detection in Tissue Engineering Southern Medical University Guangzhou Guangdong PR China; ^6^ Key Laboratory of Mental Health of the Ministry of Education Guangdong‐Hong Kong Joint Laboratory for Psychiatric Disorders; ^7^ Department of Gynecology, Zhujiang Hospital Southern Medical University Guangzhou Guangdong PR China; ^8^ Department of Biochemistry and Molecular Biology Shenzhen University Medical School Shenzhen Guangdong PR China

## Abstract

The transition from fetal primordial germ cells (PGCs) to spermatogonia (SPG) is critical for male germ cell development; however, the detailed transcriptomic dynamics and regulation underlying this transition remain poorly understood. Here by interrogating the comprehensive transcriptome atlas dataset of mouse male germ cells and gonadal cells development, we elucidated the regulatory networks underlying this transition. Our single‐cell transcriptome analysis revealed that the transition from PGCs to SPG was characterized by global hypertranscription. A total of 315 highly active regulators were identified to be potentially involved in this transition, among which a non‐transcription factor (TF) regulator TAGLN2 was validated to be essential for spermatogonial stem cells (SSCs) maintenance and differentiation. Metabolism profiling analysis also revealed dynamic changes in metabolism‐related gene expression during PGC to SPG transition. Furthermore, we uncovered that intricate cell–cell communication exerted potential functions in the regulation of hypertranscription in germ cells by collaborating with stage‐specific active regulators. Collectively, our work extends the understanding of molecular mechanisms underlying male germ cell development, offering insights into the recapitulation of germ cell generation in vitro.

## Graphical abstract

Single‐cell RNA‐seq analysis dynamic transcriptomic and regulatory networks underpinning the transition from fetal primordial germ cells to spermatogonia in mice.

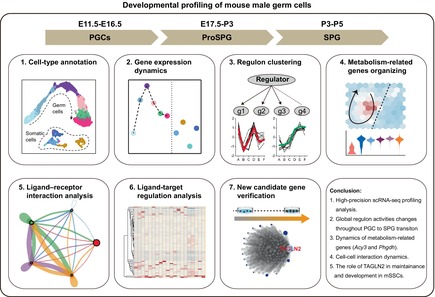

## 1 INTRODUCTION

In mammalian germ cell development, the transition from fetal primordial germ cells (PGCs) to prospermatogonia (ProSPG), followed by the differentiation of SPG/SSCs (spermatogonia/spermatogonial stem cells), constitutes one of three distinct and important phases, in addition to PGC specification and spermatogenesis.^1^ Despite the dramatic changes in the cell cycle and global epigenetic reprogramming from fetal PGCs to SPG, the sequential alterations in transcriptome are also important for cell‐fate determination and differentiation. As a direct determinant of cell‐fate, global gene expression dynamics serves as the key to unravel the differentiation of germ cell. Understanding the complex regulatory networks will not only provide insights into normal development but also has implications for infertility treatment. Thus, exploring the intricate gene expression patterns is essential for the understanding of germ cell biology.^2^, ^3^, ^4^

For human germ cell development, the relatively extensive developmental stage poses a significant challenge due to sampling gap (mainly the period before and after birth),^4^, ^5^, ^6^, ^7^, ^8^, ^9^, ^10^ making it difficult to comprehensively study the whole cell‐fate transition process. Given that the conservatism of this process between human and mouse, it would be informative to deeply explore it using mouse model. Recently we have constructed comprehensive transcriptome atlas of mouse male germ cells and gonadal cells development,^11^ and preliminarily observed distinctive gene expression patterns during fetal PGCs to ProSPG, then to SPG development. Among the candidates, we intensively explored the functions and mechanisms of certain signalling pathway and regulator, including Notch signalling pathway and HELQ. Nonetheless, a vast amount of information, such as intercellular interaction deserved in‐deep investigation by interrogating our transcriptome atlas. Additionally, in the previous attempts to recapitulate male germ cell development in vitro, low efficiency of the transition from fetal PGCs to ProSPG and then to SPG is still one of the great challenges, rendering it difficult to fulfil the whole gametogenesis in vivo.^1^, ^12^

The development of germ cells is a stepwise and precisely regulated process,^13^, ^14^ requiring the support from the surrounding somatic cell microenvironment.^15^, ^16^, ^17^, ^18^ However, the specific patterns of gene expression associated with biological events and intercellular communications, as well as the expression profiles of regulatory factors, have not been thoroughly studied. In the present study, we conducted detailed analyses of developmental and biological processes during the transition from PGC to SPG, revealing the global hypertranscription in fetal and perinatal germ cells. Subsequently, the regulators were identified throughout germ cell development, and highly active regulons specific to single stage and multiple stages were classified. We uncovered a total of 315 highly active regulators, including 135 canonical transcription factors (TFs) and 180 non‐canonical TF regulators. Among these, a novel factor, TAGLN2, was proved to be important for SSC maintenance and differentiation. Then, the metabolism profiling was analysed, and the expression patterns of phosphoglycerate dehydrogenase (PHGDH) and aminoacylase 3 (ACY3) were tracked by immunofluorescence staining, which were two candidates unreported in reproductive system. Finally, the comprehensive scRNA‐seq dataset was used to identify day‐by‐day cell–cell communication landscapes, implicating potential signalling pathways contributing to the transition from PGCs to SPG, such as COLLAGEN, AM, growth arrest‐specific gene 6 (GAS6), WNT, LAMININ, protein S (PROS), midkine (MK), as well as KIT, BMP, TGFβ and Notch. Taken together, our study revealed stage‐specific active regulators, cell–cell communication landscapes, and metabolism profiling involved in global hypertranscription during the transition from PGCs to SPG. Furthermore, we updated the mouse male germ cell landscape website at https://zhaolab2024.cpolar.top/MGC_transcriptomic_and_regulatory_networks/.

## 2 METHOD

### 2.1 Animal ethics

The germ cells and corresponding somatic cells scRNA‐seq datasets used in this study were extracted from our published atlas (publicly available in the Gene Expression Omnibus (GEO) at NCBI under accession number ‘GSE148032’) spanning from E11.5 to PND7 (embryonic day 11.5 to postnatal day 7).^11^ As previously described, male *Blimp1*‐mVenus and *Stella*‐ECFP (BVSC) transgenic mice were obtained from Mitinori Saitou laboratory (Kyoto, Japan). Female C57BL/6 mice were purchased from the Guangdong Medical Laboratory Animal Centre (Guangzhou, China). Female C57BL/6 mice in natural estrus were mated with male BVSC mice. The germ cells and somatic cells were collected using mVenus and ECFP signals ranging from E11.5 to E18.5. After birth, testicular cells were randomly selected without the assistance of FACS.

Mice were housed according to the ethical guidelines of South Medical University ethics committee (L2016149). Mouse experiments were performed in accordance with the U.S. Public Health Service Policy on Use of Laboratory Animals, and were approved by the Ethics Committee on Use and Care of Animals of Southern Medical University. Mice were kept in a standard 12 h light–dark cycle in the specific‐pathogen free conditions, and were permitted for free access to water and food. The ambient temperature was 20–25°C and the humidity was 40–70%. All the mice we used were healthy and immune‐normal.

### 2.2 Immunofluorescence staining

The mouse male gonads were fixed with 4% paraformaldehyde at 4°C overnight, and washed extensively with phosphate‐buffered saline (PBS). Then, the tissues were dehydrated and embedded in paraffin, and then cut into sections of 5‐μm (genital ridges and testis)/10‐μm (embryos) thickness, respectively. Immunostaining was performed after deparaffinization and rehydration as previously described. The primary antibodies used were mouse anti‐DDX4 antibody (1:500, Abcam #ab27591), mouse anti‐PLZF (ZBTB16) antibody (1:100, Santa Cruz Biotechnology #sc‐28,319), rabbit anti‐ETV5 antibody (1:100, Novus Biologicals #NBP2‐14950), rabbit anti‐USF1 antibody (1:100, Abcam # ab180717), rabbit anti‐ACY3 antibody (1:100, Abcam # ab197799), rabbit anti‐PHGDH antibody (1:100, Abcam # ab211365), rabbit anti‐ACY3 antibody (1:200, Abcam # ab197799), rabbit anti‐PHGDH antibody (1:400, Abcam # ab211365), rabbit anti‐TAGLN2 antibody (1:250, Abcam # ab121146), rabbit anti‐PHGDH antibody (1:100, Abcam # ab211365). The secondary antibodies used were Goat Alexa Fluor 488 anti‐rabbit IgG (1:500, Jackson ImmunoResearch #111–545‐003), Goat Alexa Fluor 594 anti‐rabbit IgG (1:500, Jackson ImmunoResearch #111–585‐003), Goat Alexa Fluor 488 anti‐mouse IgG (1:500, Jackson ImmunoResearch #115–545‐003), Goat Alexa Fluor 594 anti‐mouse IgG (1:500, Jackson ImmunoResearch #115–585‐003). Images were captured with a ZEISS LSM880 confocal microscope.

### 2.3 Quantification of immunofluorescence

Intensity profiles in each channel along highlighted yellow dotted lines in the merged images are shown (Figure 6D).

### 2.4 Mouse spermatogonial stem cell culture

Mouse spermatogonial stem cell (mSSC) lines were derived from the P5.5 testes with the genetic background of B6D2F1 (C57BL/6 × DBA). mSSCs were cultured as previously described: StemPro‐34 SFM (Invitrogen) supplemented with StemPro supplement (Invitrogen), 1% FBS, N2 (100×), 6 mg/mL D‐(1)‐glucose, 5 mg/mL BSA, 0.1 mM NEAA, 1 mM sodium pyruvate, 0.1 mM 2‐mercaptoethanol, 2 mM L‐glutamine, 10^−4^ M ascorbic acid, 10 mg/mL biotin, 30 ng/mL β‐estradiol, 60 ng/mL progesterone (Sigma), 20 ng/mL mouse EGF, 10 ng/mL human bFGF, 10 ng/mL recombinant rat GDNF (R&D Systems) and 10^3^ U/mL LIF (Merck Millipore). Cells were passaged every 6 days by dissociating with 0.05% trypsin and changed the medium every 2 days.

### 2.5 Derivation of *Tagln2* knockdown mSSCs

Two shRNAs against *Tagln2* were designed using pLKO.1‐TRC shRNA system. For lentiviral vector production, HEK293T cells cultured in 10‐cm dishes and transfect at 70–80% confluency using Lipofectamine® LTX and PLUS™ Reagent (Invitrogen) in Opti‐MEM (Thermo Fisher). After incubation for 48 h in DMEM (containing 10% FBS), the supernatant was filtered and concentrated by ultracentrifugation. Oligonucleotides used in this study were listed in the Data S9. For lentiviral transduction, mSSCs were dissociated into single cells and 2.5 × 10^4^ cells per well were cultured in a 12‐well plate. Lentivirus was added after 24 h and the medium was changed after 48 h. Puromycin was used to select transduced mSSCs at a final concentration of 0.4 μg/mL.

### 2.6 Cell proliferation, cell cycle and cell death analysis

For the cell counting assay, mSSCs were initially plated at 2.5 × 10^4^ cells per well in 12‐well plates, with passaging every 6 days, and the total cell numbers in each well were counted from passage 1 to passage 3.

The EdU proliferation assay was performed using the EdU proliferation kit (EdU Flow Cytometry Assay Kit, Yeasen) according to the manufacturer's instructions. The cells were incubated with EdU under normal cell growth conditions and fixed with 4% formaldehyde. After permeabilization, EdU‐stained cells were analysed using flow cytometer (CytoFlex, Beckman).

For analysis of cell death in knockdown mSSCs, freshly collected cells were stained with annexin V and PI (Annexin V‐FITC/PI Apoptosis Detection Kit, Keygen) and analysed using flow cytometer (CytoFlex, Beckman).

### 2.7 Quantitative PCR

Total RNA was extracted using TRIzol (TIANGEN, Y1809) Reverse transcription was performed using HiScript III Reverse Transcriptase (Vazyme, R302‐01). Quantitative PCR (q‐PCR) was conducted with the AceQ q‐PCR SYBR Green Master Mix (Vazyme, Q121‐02) on a LightCycler+96 Real‐Time PCR system (Roche). All data were normalized to the expression of housekeeping gene Gapdh and calculated by ΔCq or ΔΔCq. All q‐PCR primer pairs were listed in Data S9.

### 2.8 Single‐cell RNA‐seq data processing

Single‐cell RNA sequencing raw reads were processed to remove template switch oligo (TSO) sequence and polyA tail sequence. And then the reads with adapter and low‐quality bases were removed to obtain clean reads, The clean reads were aligned to the mm10 mouse reference using STAR^19^ (version 2.7.1a) with options ‘–outFilterMultimapNmax 3’ and ‘–outFilterMismatchNmax 4’. To count uniquely aligned reads with genes, genomic features were then added to reads in BAM file using featureCounts of subread (version 1.6.4). ‘count’ tool in UMI‐tools was used to remove PCR duplicates based on barcode and UMI information. Gene expression level was presented with log(TPM/10 + 1) unless specifically mentioned.

### 2.9 Estimation of dropout events

For each overlapped gene in germ cells between this dataset and the GSE184708 dataset, the dropout event of each gene was calculated based on the ratio of the number of cells whose gene count was zero in total number of germ cells.

### 2.10 Dimension reduction and cell‐type annotation

Seurat^20^ package (version 4.4.0) was conducted to perform dimension reduction and clustering analysis. For normal scRNA‐seq data, first, raw UMI count matrix was used to create a Seurat object. We normalized the gene expression measurements for each cell with the parameter ‘scale.factor = 100,000’. Subsequently, 4000 highly variable genes were selected to perform PCA on the scaled data. Based on ‘JackStraw’ results, top 15 significant PCs were chosen to construct a KNN graph and run non‐linear dimensional reduction (UMAP). ‘FindClusters’ function was used to cluster the cells with the parameter ‘resolution = 0.3’.

Cell types were assigned based on the expression of canonical marker genes and the differential expressed genes (DEGs) of each cluster.

### 2.11 Cell–cell communication analysis

The R package CellChat^21^ (version 1.5.0) and NicheNet^22^ (version 2.1.5) were used to characterize cell‐to‐cell interactions as previously reports.^23^, ^24^ For CellChat analysis, we divided the seurat object into subsets based on sampling different time‐points and computed the communication for each time‐point. Following the established workflow, normalized counts were uploaded into CellChat, with standard preprocessing conducted using the identifyOverExpressedGenes, identifyOverExpressedInteractions and projectData functions with specific parameters. Potential ligand‐receptor interactions were then calculated using the computeCommunProb, computeCommunProbPathway and aggregateNet functions with standard parameters. For NicheNet analysis, Germ cells were categorised into PGCs, ProSPG and SPG. We defined germ cell as receiver cells, and the somatic cells of the corresponding time‐points as sender cells. Nichenet_seuratobj_aggregate function was used to connect the results of Seurat with the analysis of NicheNet.

### 2.12 Single‐cell regulatory network inference and clustering analysis

The single‐cell regulatory network inference and clustering (SCENIC)^25^ pipeline (as implemented in pySCENIC) was performed to infer TFs, gene regulatory networks (GRN) of germ cell development as previously described.^26^, ^27^ The co‐expression network was calculated by runGenie3 and the regulons were identified by RcisTarget. The regulon activity for each cell was scored by AUCell.

### 2.13 Weighted gene co‐expression network analysis

The R package high‐dimensional weighted gene co‐expression network analysis (hdWGCNA)^28^ was used to identify pivotal module genes involved in germ cell. First, we employed the setup for WGCNA function to construct an expression matrix from the Seurat object. Subsequently, using the MetacellsByGroups function, we built averaged ‘metacells’ based on cell types (with 20 nearest neighbour cells and a maximum shared cell count of three between two metacells). Following this, we normalized and standardized the metacell matrix through various means. Next, we used the SetDatExpr function to set the expression matrix and the TestSoftPowers function to calculate the topological indicators of the network, assisting in the selection of the optimal soft threshold. Ultimately, the ConstructNetwork function was utilized to establish a co‐expression network, and the ModuleEigengenes function was used to compute the ‘eigenvectors’ of the modules to obtain module feature genes.

### 2.14 Self‐organizing map analysis

The kohonen package in R was used to train a self‐organizing map (SOM) for 3838 metabolism related genes (downloaded from https://www.gsea-msigdb.org/gsea/msigdb). UMI count was first normalized to TPM and median value was calculated for each type of germ cells to generate a gene‐cell type matrix, which is used as the input data for SOM training. The total number of map units was set to 100. Visualization was performed with custom R code.

### 2.15 RNA‐seq library construction and sequencing

Total RNA was extracted from sh‐NC and sh‐*Tagln2* mSSCs using TRIzol Reagent (Ambion). 5 ng total RNA per sample was purified through poly(A) selection and subsequently utilized for library construction using the STRT‐seq method with 3′ terminal cDNAs enrichment. Sequencing was performed on NovaSeq‐PE150.

### 2.16 Statistical analysis

The experimental data was statistically analysed using unpaired two‐tailed t test to compare differences between different groups with GraphPad Prism.

## 3 RESULTS

### 3.1 A single‐cell transcriptome map during fetal PGCs to SPG transition

**FIGURE 1:**
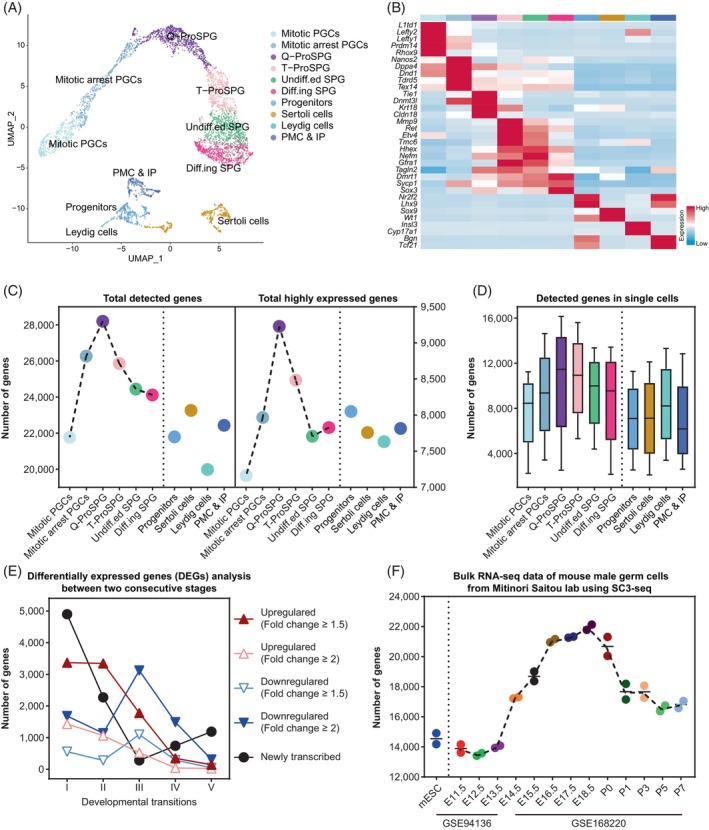
A single‐cell transcriptome map during fetal PGCs to SPG transition. (A) UMAP (Uniform Manifold Approximation and Projection) plot of the mouse germ cells and somatic cells during the fetal PGC and SPG stages. Cells are coloured by indicated cell types. PMC and IP, peritubular myoid cells and interstitial progenitors. (B) Heatmap of representative marker genes. The colour key from blue to red indicates low to high expression levels. PMC and IP, peritubular myoid cells and interstitial progenitors. (C) The total number of detected genes (left) or highly expressed genes (right) in each cell types. (D) The number of genes detected in single cell of each cell types. (E) DEGs analysis of two consecutive cell types in germ cells. For newly transcribed genes, expression was 0 in the previous stage, but expression was detected in the subsequent stage. (F) The number of genes detected in germ cells across the time‐points by bulk RNA‐seq. Data of mESCs were used as control. DEGs, differential expressed genes; IP, interstitial progenitors; PGCs, primordial germ cells; PMC, peritubular myoid cells; SPG, spermatogonia.

To investigate the dynamic transcriptome changes and molecular events characterizing the transition from fetal PGCs to SPG, we extracted the germ cells and corresponding somatic cells covering this period from our published atlas spanning from E11.5 to PND7 (embryonic day 11.5 to postnatal day 7).^11^ Dimensional reduction and clustering analysis were performed to group cells according to the global gene expression signatures (Figure 1A). As expected, the distribution of germ cells aligns along the developmental trajectory, predominantly clustering into six groups, including mitotic PGCs, mitotic arrest PGCs, quiescent ProSPG (Q‐ProSPG), transitional ProSPG (T‐ProSPG), undifferentiated SPG (Undiff.ed. SPGs) and differentiating SPG ( Diff.ing SPG).^7^, ^9^, ^17^ Referring to the traditional naming system^16^, ^17^, ^29^ (Table S1), mitotic PGCs correspond to multiplying ProSPG (M‐ProSPG); mitotic arrest PGCs and Q‐ProSPG constitute primary transitional ProSPG (T1‐ProSPG); T‐ProSPG includes both intermediate ProSPG (I‐ProSPG ) and secondary transitional ProSPG (T2‐ProSPG). These germ cell groups exhibited distinct expression patterns of marker genes (Figure 1B), such as *Prdm14* ^30^ in mitotic PGCs and *Nanos2* ^31^ in mitotic arrest PGCs. As showed in the heatmap, T‐ProSPG and Undiff.ed. SPG had considerable overlap in marker gene expression, including *Gfra1*, alongside the newly identified genes like *Hhex* and *Tagln2*.^32^ In contrast, Diff.ing SPG had elevated expression of differentiation‐associated genes, such as *Dmrt1*.^33^ Meanwhile, four somatic cell types were identified as progenitor cells, peritubular myoid cells (PMC) and interstitial progenitors (IP), leydig cells and sertoli cells, respectively. The proportion of these cell types across development was also recorded (Figure S1A).

As demonstrated above, our single‐cell transcriptome sequencing dataset provided comprehensive coverage of both time‐points and cell types during fetal PGC to SPG. We then assessed the quality of our data and found that, compared to data generated using 10× genomics sequencing, our data from single‐tube amplification‐based libraries exhibited significant advantages in terms of gene detection rate and dropout rate^34^, ^35^ (Figure S1B,C), which would be conductive to the following in‐depth data mining in this study.

### 3.2 The transition from PGCs to SPG is characterized by global hypertranscription

Utilizing our high‐precision continuous dataset, we found that the number of genes detected in germ cells dynamically changes during development (Figure 1C). Notably, the highest gene expression is detected at the Q‐ProSPG, regardless of whether the threshold is ‘detected (log(TPM/10 + 1) >0)’ or ‘highly expressed (log(TPM/10 + 1) >1)’ (Figure 1C). Additionally, the Q‐ProSPG also has the highest number of genes detected per single cell (Figure 1D). Next, we analysed DEGs from mitotic PGC to T‐ProSPG, and observed a substantial increase in the number of upregulated genes, especially during transition I (from mitotic PGCs to mitotic arrest PGCs) and transition II (from mitotic arrest PGCs to Q‐ProSPG) (Figure 1E). GO term analysis revealed that these upregulated genes are highly related to reproductive processes, general developmental processes and biological processes and regulation (Data S1). We then validated our findings using published data from bulk RNA‐seq of mouse male germ cells and found that the dynamic changes in transcriptional activity in germ cells were confirmed (Figure 1F and Figure S1D). Furthermore, based on the expression of housekeeping genes and germ cell‐specific expressed genes, as well as the detection of genes in somatic cells (Figure S1E,F), we demonstrated that these results were not due to single‐cell sequencing technology bias or sampling bias.

Furthermore, we examined the dynamic changes in gene sets associated with several classical biological processes (Figure S1G and Data S1). These distinct patterns demonstrated that our data can sensitively capture the characteristics of the cell types using a specific gene set, primarily based on the relative expression levels and the number of highly expressed genes. Importantly, consistent with the aforementioned findings, the ProSPG consistently captures a greater number of genes.

In conclusion, we observed a prominent change of global transcriptional activity in fetal and perinatal germ cells with day‐by‐day resolution for the first time. Previous report noted a global hyperactivity of the germline transcriptome at E13.5 and E15.5.^36^ However, in our study, we found that the hypertranscription in E16.5‐P7 germ cells is more pronounced and intense (Figure 1F and Figure S1D).

### 3.3 Hypertranscription is companied by global regulon dynamics in germ cells

Cell‐fate transition is highly associated with the transcriptional state, which always arises from an underlying GRN.^25^ Within this network, a set of canonical TFs and non‐canonical regulators, such as cofactors, enzymes and other regulators, mutually regulate one another along with their downstream target genes. The regulators and their target genes constitute subnetworks, referring to ‘regulons’, are capable of defining cell‐fate. Based on the transcriptome dynamics described above, we asked whether hypertranscription in germ cells were driven by TFs and other regulators. To find the candidate regulators governing the transition from fetal PGCs to ProSPG, then to SPG, we performed single‐cell regulatory network inference and clustering (SCENIC) analysis to score the activity of regulons.

**FIGURE 2:**
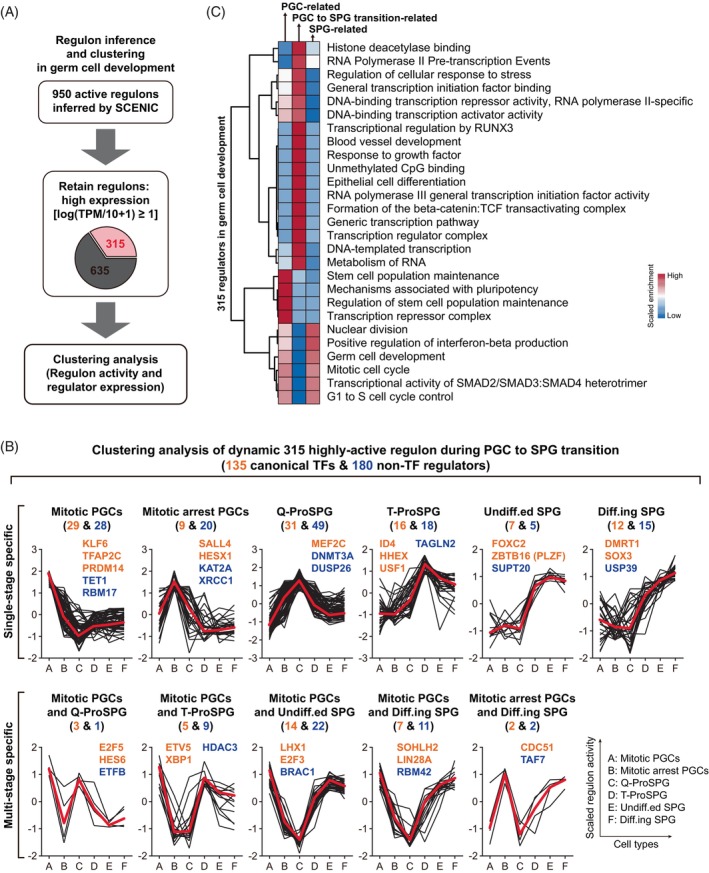
Hypertranscription is companied by global regulon dynamics in germ cells. (A) SCENIC regulon analysis workflow. (B) Clustering analysis of dynamic regulon during PGC to SPG transition. Eleven modules of regulons form two categories according to the regulon activity changing patterns. Regulon numbers per cluster were shown (orange for canonical TFs, and dark blue for non‐TF regulators). Mean of scaled regulon activity level of each module (red line) was shown. (C) Representative GO terms (performed by Metascape tool with well‐adopted hypergeometric test and Benjamini‐Hochberg *p*‐value) of the 315 highly active regulators in germ cell development. PGCs, primordial germ cells; SCENIC, single‐cell regulatory network inference and clustering; SPG, spermatogonia; TFs, transcription factors.

Based on the SCENIC results, 315 relatively highly active regulons were identified, including 135 canonical TFs and 180 non‐TF regulators (Figure 2A and Data S2). To delineate the step‐wised regulator regulation landscape, regulon activity dynamics of six types of germ cells were examined (Figure 2B). Eleven modules of regulons with distinct activity changing patterns were classified into two categories among these consecutive types of germ cells. Category I represented regulons were specifically active mainly in a single stage, such as TFAP2C regulon in mitotic PGCs and non‐TF regulator TAGLN2 in T‐ProSPG. While those were active in multiple inconsecutive stages were involved in category II, such as LHX1 regulon in both mitotic PGCs and Undiff.ed. SPG (Figure 2B). Among these, the expression of two uncharacterized factors, ETV5 and USF1, were tracked by immunostaining (Figure S2).

Transcription is generally driven by the TFs and their associated regulators, as well as the transcription machinery. Through SCENIC analysis, we found that there were the highest number of active regulons in ProSPG, consistent with the hypertranscription in germ cells. Next, we conducted enrichment analysis on these regulators to elucidate their roles (Figure 2C). The active regulators in mitotic arrest PGC, Q‐ProSPG and T‐ProSPG, which are PGC to SPG transition‐related regulators, were more specifically involved in transcription processes compared to PGC‐related and SPG‐related regulators, including ‘generic transcription pathway’, ‘DNA‐templated transcription’, ‘RNA polymerase II pre‐transcription events’ and ‘epithelial cell differentiation’ (Figure 2C). While other regulators are involved in ‘germ cell development’, ‘mitotic cell cycle’ and ‘stem cell population maintenance’. These results indicated that hypertranscription in germ cells is driven by the stage‐specific active TFs and non‐TF regulators, orchestrating the global transcriptome reconfiguration in germ cells.

### 3.4 Metabolism profiling during the PGC to SPG transition

**FIGURE 3:**
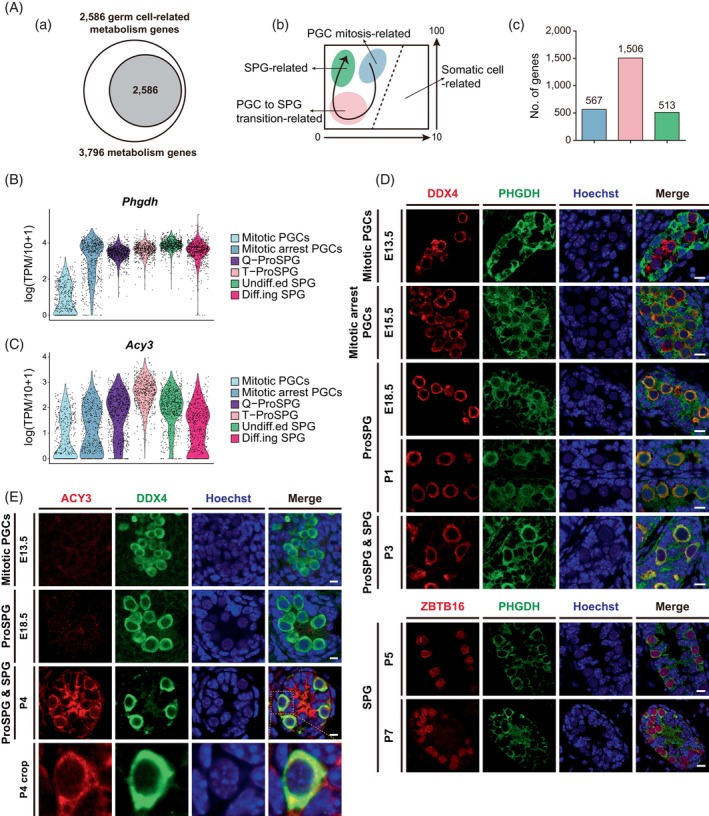
Metabolism profiling during the PGC to SPG transition. (A) Results of self‐organizing map algorithm (SOM) analysis: (a) Pie chart showing the distribution of germ cell related metabolism genes in the total metabolism profiles. (b) Schematic showing the co‐expression patterns during PGC to SPG transition related to the SOM results above. (c) Barplot showing the numbers of PGC‐related, PGC to SPG transition‐related and SPG‐related metabolism genes in the SOM results. (B) Violin plots showing the relative expression levels (log(TPM/10 + 1)) of *Phgdh* across mitotic PGCs to Diff.ing SPG. (C) Violin plots showing the relative expression levels (log(TPM/10 + 1)) of *Acy3* across mitotic PGCs to Diff.ing SPG. (D) Immunofluorescence of PHGDH (green) co‐stained with DDX4 or ZBTB16 (red) and Hoechst (blue) in mouse male gonads. Scale bar, 10 μm. (E) Immunofluorescence of ACY3 (red) co‐stained with DDX4 (green) and Hoechst (blue) in mouse male gonads. Scale bar, 10 μm. ACY3, aminoacylase 3; PGCs, primordial germ cells; PHGDH, phosphoglycerate dehydrogenase; SPG, spermatogonia.

Cell‐fate transition during gonadal germ cell and somatic cell development involves the participation of metabolism‐related genes.^37^, ^38^ However, the expression dynamics of these genes in the context of the PGC to SPG development is hitherto unknown. To this end, metabolism‐related genes were clustered by the SOM, and we found that each cell cluster enriched different sets of metabolism‐related genes (Figures 3A and S3A and Data S3). Through single‐cell transcriptomic profiling, we identified that 2586 out of 3795 metabolism‐related genes were highly enriched in germ cells. These genes were subsequently classified into three categories based on co‐expression patterns: PGC mitosis‐related, PGC to SPG transition‐related and SPG‐related. Interestingly, the largest number of metabolism‐related remains PGC to SPG transition‐related, consistent with the aforementioned hypertranscription.

PGC mitosis‐related genes were enriched in GO terms (Figure S3B) such as ‘cell cycle’, ‘organophosphate biosynthetic process’, ‘regulation of chromosome organization’, ‘DNA replication’ and ‘cellular responses to stress’, indicating their association with the active mitosis state of PGCs.^37^, ^39^ Metabolism‐related genes involved in the PGC to SPG transition were enriched in GO terms such as ‘monatomic ion transmembrane transport’, ‘transport of small molecules’, ‘monocarboxylic acid metabolic process’, ‘metabolism of lipids’ and ‘biological oxidations’. These metabolic events, primarily occurring in ProSPG, facilitate the transition from PGC to SPG. In contrast, genes highly expressed in SPG were enriched in biological processes such as ‘purine metabolism’, ‘cell cycle, mitotic’, ‘amino acid metabolic process’, ‘glutathione and one‐carbon metabolism’ and ‘l‐serine biosynthetic process’. These pathways indicated the transition from a quiescent state to active cell cycling, relying on critical metabolic pathways involving purine, amino acids and glutathione.

To validate the metabolism predictions generated by scRNA‐seq at the protein level, we selected markers previously unreported in reproductive systems. We examined the expression pattern of PHGDH (Figure 3B,D), which played a critical role in the early steps of L‐serine synthesis. Consistent with the mRNA expression pattern, PHGDH was barely detectable in germ cells at E13.5 but highly expressed in ProSPG and SPG, suggesting its role in regulating the quiescent state and re‐entry into mitosis. This may also relate to the switch in energy metabolism pathways, with germ cells relying less on aerobic respiration post‐colonization into genital ridge.^39^ Additionally, PHGDH might function as a sensor for 3‐phosphoglycerate,^40^ thereby regulating short‐term cell‐fate. Similarly, we observed the expression pattern of ACY3 in germ cells, which was involved in biological oxidations^41^, ^42^ (Figure 3C,E).

### 3.5 Cell–cell interaction analysis during fetal to postnatal stages

Germ cell development and differentiation are fundamental biological processes facilitated by intricate cell–cell interactions and communications within the microenvironment of the gonads. However, the signalling pathways that drive the transition of PGCs into SPG remain to be elucidated. Besides, we were interested in investigating whether intercellular communication contributes to hypertranscription in germ cells. Here, we have reconstructed a day‐by‐day single‐cell molecular interaction map spanning from fetal to postnatal stages.

**FIGURE 4:**
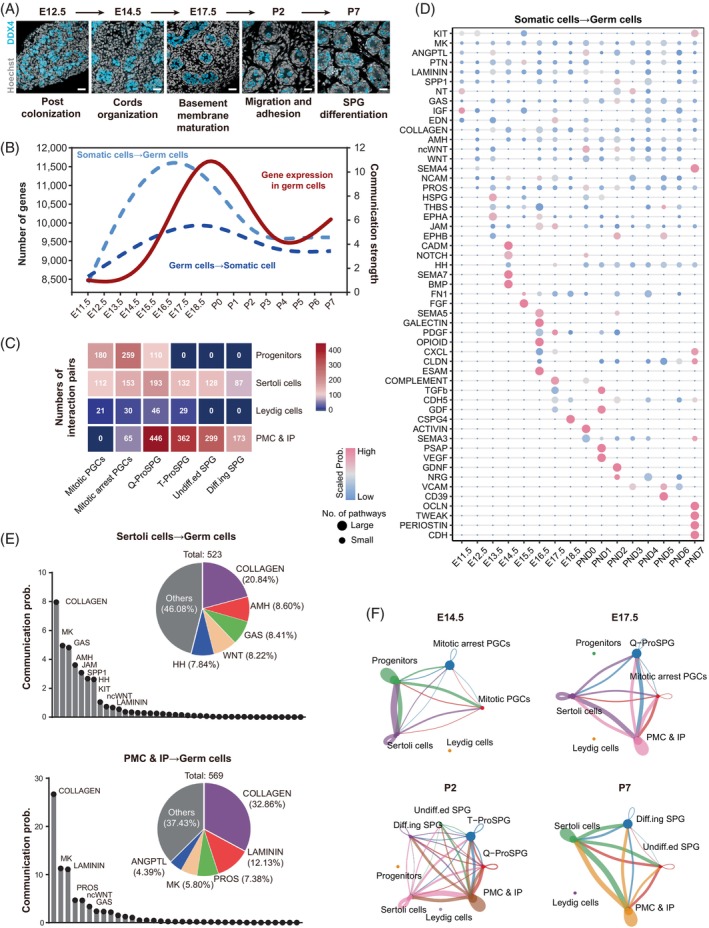
Cell–cell interaction analysis during fetal to postnatal stages. (A) Representative sections of mouse male gonadal structure across development. Scale bar, 10 μm. (B) Number of genes detected in each germ cell types (fitted from Figure S1D), and cell–cell communication strength across the time‐points. (C) Numbers of interaction pairs between germ cells and somatic cells. (D) Scales probabilities and numbers of signalling pathways from somatic cells to germ cells across the time‐points. (E) Top: Summary and statistics of signalling pathways from Sertoli cells to germ cells. Bottom: Summary and statistics of signalling pathways from PMC and IP to germ cells. (F) Circle plots of cell–cell communications at E14.5, E17.5, P2 and P7. IP, interstitial progenitors; PMC, peritubular myoid cells.

After colonizing the genital ridge, PGCs and fetal gonadal somatic cells are relatively interspersed.^43^ After E12.5, male germ cells were surrounded by somatic cells to form cord (Figure 4A). Interestingly, upon observing the communication strength between germ cells and somatic cells, we found that interactions were most intense from E14.5 to P1 (Figure 4B). Integration of the temporal trends of intercellular communication intensity and the number of detected genes in germ cells suggested that intercellular communication signals from somatic cells to germ cells significantly contribute to hypertranscription in germ cells (Figure 4B).

This period from E14.5 to P1 coincides with the formation of cords and the maturation of the basement membrane, indicating that the differentiation of PGCs into SPGs was closely associated with the maturation of testicular structure.^44^ To further investigate how somatic cells participated in germ cell differentiation, we analysed the intercellular interactions between germ cell and somatic cell. Notably, we found that the interaction intensity gradually changed alongside the developmental process, as seen from germ cells↔progenitors interactions to germ cells↔Sertoli cells and finally became germ cells↔PMC and IP interactions (Figure 4C). Next, the detailed day‐by‐day interaction strength and numbers of sub‐pathways involved in ‘cell–cell contact’, ‘extracellular matrix(ECM)‐receptor’ and ‘secreted signalling’ were arranged (Figures 4D and S4A and Data S4). Consistent with previous studies,^11^, ^45^, ^46^, ^47^, ^48^ we observed dynamic changes in signalling pathways such as KIT, BMP, TGFβ and Notch during development. Then we summarized the vast and intricate signalling pathways involved in these interactions (Figure 4E). The key signalling pathways include: for Sertoli cells→germ cells, COLLAGEN,^49^ AMH,^50^, ^51^ GAS (growth arrest‐specific gene 6, GAS6),^52^, ^53^ WNT,^54^, ^55^ junctional adhesion molecule (JAM),^56^, ^57^ secreted phosphoprotein 1 (SPP1)^58^ and hedgehog (HH).^59^, ^60^ For PMC and IP → germ cells, COLLAGEN,^61^ LAMININ,^62^ PROS, MK^63^ and angiopoietin‐like protein (ANGPTL). Additionally, cell–cell interaction networks were constructed in a daily resolution (Figure 4F and Figure S4B), and the detail data could be found in Data S4.

### 3.6 Potential association between gonadal somatic cells and hypertranscription in germ cells

**FIGURE 5:**
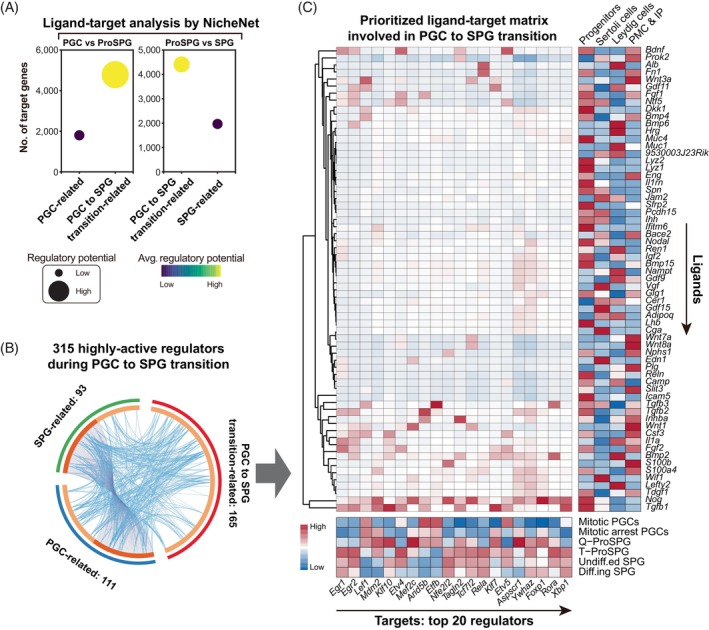
Potential association between gonadal somatic cells and hypertranscription in germ cells. (A) The number of target genes and regulatory potential in germ cells upon the regulation from ligands in gonadal somatic cells. (B) Circos plot showing the 315 highly active regulators from Figure 2, PGC to SPG transition‐related genes represent regulators highly expressed in Q‐ProSPG and T‐ProSPG. (C) Prioritized ligand‐target matrix between gonadal somatic cells (ligand) and germ cells (target) predicted by NicheNet. The top 20 regulators are ranked by the corresponding total regulatory potential from somatic cells. The expression level of the ligands and targets were also shown. IP, interstitial progenitors; PGCs, primordial germ cells; PMC, peritubular myoid cells; ProSPG, prospermatogonia; Q‐ProSPG, quiescent ProSPG; SPG, spermatogonia; T‐ProSPG, transitional ProSPG.

Given that the ascending temporal trends in intercellular communication intensity (initially) and the number of detected genes in germ cells (subsequently) (Figure 1 and Figure 4B), it is reasonable that cell–cell interactions signalling from gonadal somatic cells might participate in the regulation of hypertranscription in germ cells. To test this hypothesis, ligand‐target activity was predicted using NicheNet^22^ (Figure 5A and Data S5). The regulatory network connected the ligands in somatic cells and targets in germ cells at the corresponding time‐points.

To simplify analysis, we combined mitotic PGCs and mitotic arrest PGCs as PGCs, combined Q‐ProSPG and T‐ProSPG as ProSPG, and combined undiff.ed. SPG and diff.ing SPG as SPG. Then, comparisons of ligand‐target activities between ProSPG versus PGCs and SPG were conducted, respectively (Figure 5A and Data S5). These two profiles consistently showed that ProSPG exhibited the strongest ligand‐target activity. Meanwhile, targets regulated by somatic cell‐derived ligands were notably abundant, and the average regulatory potential per target was higher in ProSPG. These findings suggested a significant role of gonadal somatic cells in driving hypertranscription in germ cells.

Next, we examined the regulatory status of 315 regulators uncovered above (Figure 5B). The prioritized ligand‐target matrix highlighted the top 20 regulators relevant to the transition from PGC to SPG (Figure 5C). For instance, *Tagln2* was found to be regulated by members of the TGF‐beta superfamily (*Tgfb1* and *Inhba*), the transmembrane receptor protein serine/threonine kinase signalling pathway (*Nog*), and the nerve growth factor family (*Bdnf*) from somatic cells (Figure 5C). Thus, the intercellular interaction between gonadal somatic cell and germ cell might also involve in the regulation of hypertranscription in germ cells.

### 3.7 TAGLN2 is involved in SSC maintenance and differentiation

**FIGURE 6:**
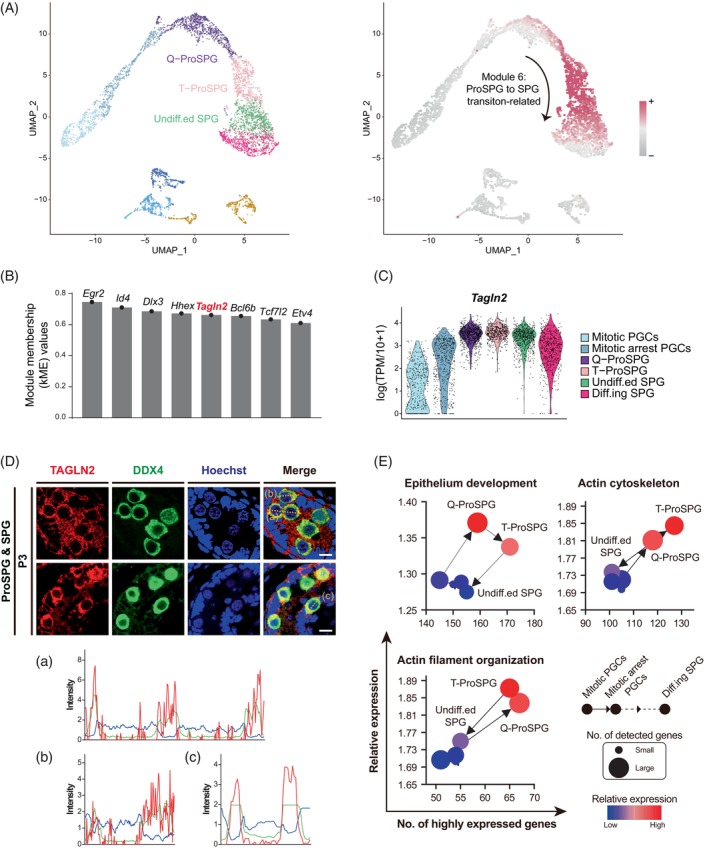
TAGLN2 is predicted as a hub regulator in the ProSPG to SPG transition. (A) Feature plot of co‐expression genes in module 6. (B) Module membership (kME) values of top regulators related to ProSPG to SPG transition. (C) Violin plots showing the relative expression levels (log(TPM/10 + 1)) of *Tagln2* across mitotic PGCs to Diff.ing SPG. (D) Top: Immunofluorescence of TAGLN2 (red) co‐stained with DDX4 (green) and Hoechst (blue) in mouse male gonads. Scale bar, 10 μm. Bottom: Intensity profiles in each channel along highlighted yellow dotted lines in the merged images above are shown. (E) Dynamics of *Tagln2*‐related gene sets. PGCs, primordial germ cells; ProSPG, prospermatogonia; SPG, spermatogonia.

As above mentioned, key non‐TF regulators were also identified in SCENIC analysis, with an indirect or collaborative effect in the regulation networks. Among these 180 non‐TF regulators, *Tagln2* caught our attention for its high regulon activity and potential roles in ProSPG to SPG transition (Figure 2B). Then, WGCNA also confirmed that *Tagln2* was the fifth hub regulator during ProSPG to SPG transition (Figures 6A,B and S5 and Data S6). Interestingly, *Tagln2* was the only non‐TF regulator in the top hub genes. *Tagln2*, encoding a actin‐binding protein, is involved in actin filament, and actin cytoskeleton organization during cell invasion, cell proliferation, cell migration and epithelial cell differentiation.^64^, ^65^, ^66^, ^67^ As a marker of differentiated smooth muscle, we wondered whether *Tagln2* played critical roles in germ cell development (Figure 6C,D). To this end, *Tagln2*‐related biological processes were examined during cell development, and the results showed that epithelial cell differentiation and actin organization were highly active in ProSPG and SPG (Figure 6E).

**FIGURE 7:**
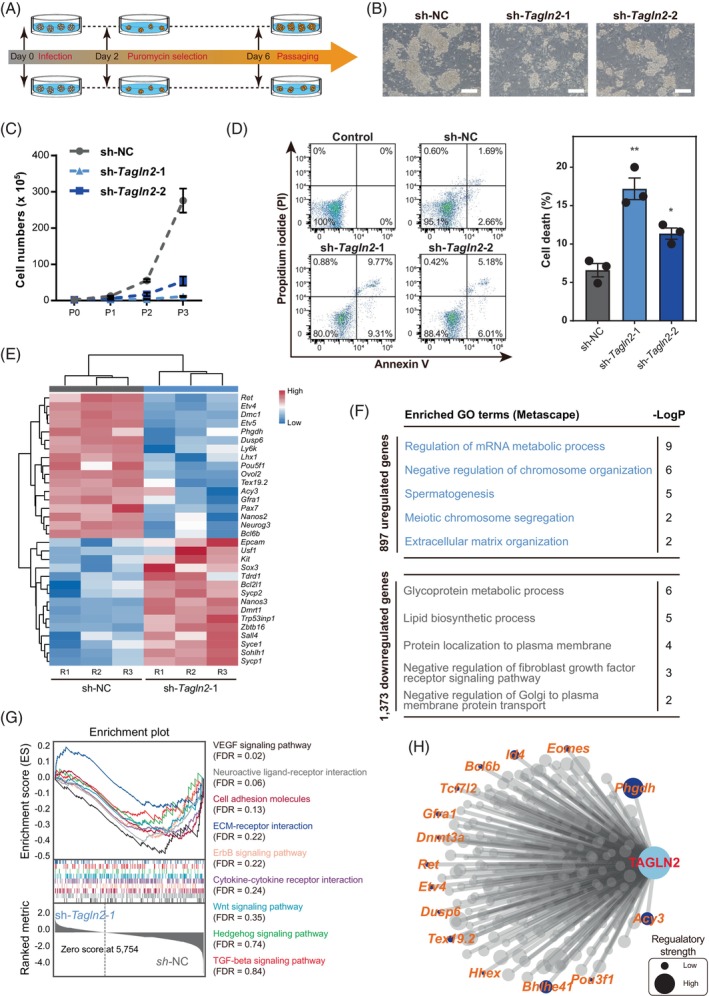
TAGLN2 is involved in SSC maintenance and differentiation. (A) Schematic showing the workflow of gene knockdown in mSSCs. (B) Representative cell morphology of two *Tagln2*‐knockdown mSSC lines (sh‐*Tagln2*‐1 and sh‐*Tagln2*‐2) compared to control mSSCs transduced with non‐targeting shRNA (sh‐NC). Scale bar, 200 μm. (C) Proliferation of sh‐NC, sh‐*Tagln2*‐1 and sh‐*Tagln2*‐2 mSSC lines. Data are shown as the mean ± SEM, *n* = at least 3 biologically independent samples for each group. (D) Flow cytometry analysis of cell death in sh‐NC‐, sh‐*Tagln2*‐1 and sh‐*Tagln2*‐2‐transfected mSSCs through double staining with annexin V and PI. Annexin V^+^ PI^−^ cells, the indicator of early‐stage apoptotic cells; Annexin V^+^ PI^+^ cells, the indicator of late‐stage apoptotic cells and other kinds of dead cells. Quantitative data are shown as the mean ± SEM to the right, *n* = 3, *p < 0.05, **p < 0.01, unpaired two‐tailed t test. (E) Heatmap showing the differentially expressed marker genes related to SSC maintenance and differentiation. The colour key from blue to red indicates low to high expression levels. (F) Enriched GO terms of the upregulated and downregulated genes in sh‐*Tagln2*‐1 mSSCs. (G) Enrichment plots of KEGG pathways involved in cell–cell interaction upon *Tagln2* knockdown. (H) Inferred TAGLN2 regulation network visualizing potential key regulators from SCENIC and *Tagln2* knockdown results. Selected top nodes are coloured in dark blue and labelled in orange. mSSC, mouse spermatogonial stem cell; SCENIC, single‐cell regulatory network inference and clustering.

To further explore whether *Tagln2* participated in the regulation of survival, maintenance and differentiation of germ cells, we knocked down *Tagln2* in mSSCs (Figure 7A,B and Figure S6A). *Tagln2* knockdown impaired the mSSC clone formation and decreased cell proliferation (Figure 7C and Figure S6B,C). Meanwhile, knockdown of *Tagln2* promoted the cell death of mSSCs (Figure 7D). Genome‐wide transcriptome was then performed (Figure S6D). We found that *Tagln2* knockdown resulted in low expression of marker genes related to SSC maintenance and pluripotency (Figure 7E), such as *Etv4*, *Etv5*, *Ret*, *Gfra1*, *Lhx1*, *Neurog3*, *Bcl6b* and *Pou5f1*.^68^, ^69^, ^70^, ^71^, ^72^, ^73^, ^74^ Interestingly, *Phgdh* and *Acy3* were also downregulated upon *Tagln2* knockdown (Figure 7E), implying the roles related to SSC maintenance of these two metabolism markers. In contrast, genes related to SPG differentiation were aberrantly upregulated, consistent with the fact that mSSC could not be maintained after *Tagln2* knockdown. Next, the genes abnormally upregulated after *Tagln2* knockdown were enriched in spermatogenesis, meiotic chromosome segregation and extracellular matrix organization, while the abnormally downregulated genes were involved in glycoprotein metabolic processes (Figure 7F and Data S7). Then, signalling pathways related to cell–cell interactions and communications involved in germ cell development were analysed upon *Tagln2* knockdown (Figure 4D and Figure 7G). *Tagln2* knockdown significantly impaired cell–cell interaction networks, such as ‘VEGF signalling pathway’, ‘Neuroactive ligand‐receptor interaction’, ‘Cell adhesion molecules’, ‘ECM‐receptor interaction’, ‘ErbB signalling pathway’ and ‘Cytokine‐cytokine receptor interaction’. Finally, *Tagln2* regulation network was constructed in the context of ProSPG to SPG transition based on these results (Figure 7H and Data S8). Together, TAGLN2 was identified as a key regulator involved in male germ cell survival, maintenance and differentiation.

## 4 DISCUSSION

**FIGURE 8:**
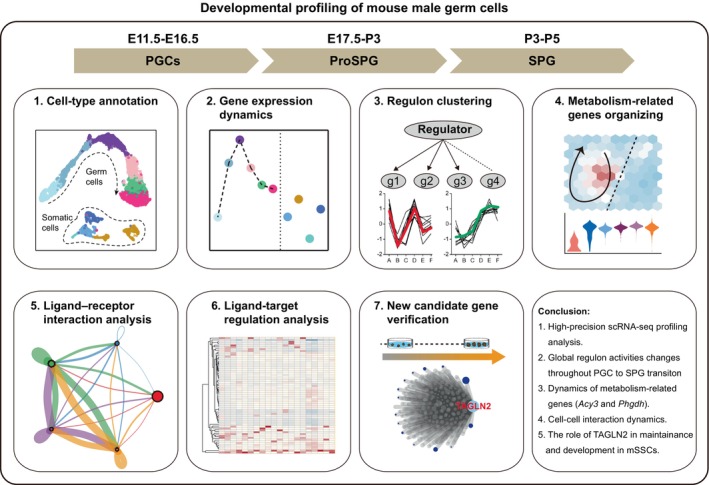
Workflow of scRNA‐seq analysis revealing transcriptomic dynamics of mouse male germ cells during the fetal to postnatal stages. PGCs, primordial germ cells; ProSPG, prospermatogonia; SPG, spermatogonia.

In this study, we investigated the dynamic transcriptome changes and molecular events inherent to the transition from fetal PGCs to SPG in mice (Figure 8). By interrogating the single‐cell transcriptome data across developmental stages (E11.5 to PND7), we offer several key insights into male germ cell development and differentiation.

First, our dataset exhibited comprehensive coverage and high‐quality. We performed an analysis of developmental and biological processes, revealing dynamic changes in gene expression. To the best of our knowledge, this was the first to describe such dramatic and dynamic changes in transcriptional activity and hypertranscription in fetal and perinatal germ cells with day‐by‐day resolution. Previous reports found that E13.5 and E15.5 germ cells were more transcriptionally active compared with somatic cells^36^; however, global hypertranscription was detected to be more pronounced in E16.5‐P7 germ cells.

Second, analysis of canonical TF and non‐canonical TF regulons identified key regulators associated with specific stages of germ cell development. The differential activity of regulons across developmental stages suggested stage‐specific regulatory programs governing germ cell‐fate determination. Our results indicated that hypertranscription during PGC to SPG transition was driven by the stage‐specific active TFs and non‐TF regulators, providing valuable insights into the transcriptional control of germ cell development.

Additionally, metabolism profiling analysis revealed distinct metabolic gene expression patterns during the transition from PGCs to SPG. Through immunofluorescence tracking of PHGDH and ACY3 expression patterns, we identified previously undescribed markers of metabolism in mouse male gonads. PHGDH's role in serine biosynthesis and one‐carbon unit production is well‐documented,^75^, ^76^ while the function and mechanism of PHGDH in germ cells remain unexplored. Our study revealed its dynamic expression during PGC to SPG differentiation, laying the groundwork for future functional study. Given that global PHGDH knockout results in embryonic lethality,^77^ Cre‐loxP system should be used for conditional genetic ablation in germ cells. Interestingly, PHGDH shows high expression in early gonadal somatic cells but decreased expression around birth. ACY3 is primarily involved in the hydrolysis of N‐acetylated amino acids into free amino acids and acetic acid, contributing to cellular processes such as protein metabolism and detoxification pathways.^41^, ^42^, ^78^ In germ cells, the potential functions of ACY3 have not been extensively studied. Given its known functions in other tissues,^78^ ACY3 might help regulate the availability of free amino acids by hydrolyzing N‐acetylated amino acids, thus supporting the high metabolic demands of developing germ cells. But the detailed functions and mechanisms of PHGDH and ACY3 in gonadal development warrant further investigation.

Furthermore, investigation of cell–cell interactions highlighted the potential roles and significance of gonadal somatic cells associated with hypertranscription in germ cells. Dynamic communication between germ cells and somatic cells, particularly during the formation of cords and the maturation of the basement membrane, underscores the intricate interplay between cell types during gonadal development. These findings will help understand the temporal dynamics of male germ cell development. Although our mapping aims to closely reflect the actual developmental timeline of cell types, spatial transcriptomic analysis is need to create a comprehensive spatiotemporal network of cell–cell communication during development.

Finally, we identified TAGLN2 to be a key regulator involved in germ cell development, analysis and experimental results highlighted the functional significance of TAGLN2 in SSC maintenance and differentiation. The mechanisms by which ProSPG to SPG transition and SSCs migrate and differentiate in the seminiferous tubules are still unclear. Genes regulating the cytoskeleton play important roles in cell deformation and migration, and they also participate in regulating basic functions that maintain cell survival. In this study, we used TAGLN2 as an example to explore the function of such genes, proving that TAGLN2 plays an important role in maintaining mSSC proliferation and survival. Previous studies have shown that TAGLN2 also participates in cell migration, but whether TAGLN2 also plays a similar role in germ cells is unknown. It is also worth investigating whether the function and mechanism of TAGLN2 in ProSPG to SPG transition and SPG differentiation. For example, different conditional knockout strategies can be used to knock out *Tagln2* in ProSPG (using *Ddx4*‐cre^79^) and SPG (using *Stra8*‐cre^80^), respectively.

It is well‐known that global DNA demethylation of mouse male germ cells occurs between E11.5 and E13.5, during which the overall methylation level drops below 20%.^81^ Cells at this stage are mitotic PGCs, corresponding to M‐ProSPG. Interestingly, mitotic PGCs do not exhibit obvious hypertranscription when compared to later mitotic arrest PGCs and Q‐ProSPG (T1‐ProSPG). This suggests that global DNA demethylation might settle a permissive chromatin state prior to hypertranscription.^36^ It also raises questions about whether de novo methylation in the retrotransposon regulatory region are related to hypertranscription,^82^, ^83^ and whether the reprogramming of histones such as H3K27me3, H3K9me2/3 and H3K36me2/3 is also associated with this process.^83^, ^84^, ^85^, ^86^, ^87^ One hypothesis is that these active retrotransposon regulatory elements might function as enhancers driving hypertranscription, but it warrant further in‐depth research in the future.

In conclusion, the present study extends our understanding of the molecular mechanisms governing the transition from fetal PGCs to SPG. By integrating single‐cell transcriptomic data with functional validation experiments, this study provides new insights into germ cell development and identify potential targets for future research and therapeutic interventions.

## AUTHOR CONTRIBUTIONS

G.C., X.‐Y.Z. and J.Z. conceived and supervised the project. J.Z., M.C., C.W., Z.L., Y.Z. and M.W. performed the experiments. With the help of K.T., G.J., X.Y. and Z.O. performed bioinformatics analysis. J.Z., G.C., X.‐Y.Z., K.T. and G.J. wrote the manuscript with the help from all the authors.

## CONFLICT OF INTEREST STATEMENT

The authors declare no conflict of interest.

## Supporting information


**Figure S1.** related to Figure 1: (A) Proportion of germ cell types across the time‐points. (B) Boxplot showing the number of detected genes per cell in this dataset and GSE184708. Centre line, median; box limits, interquartile range (IQR); whiskers, minima and maxima within 1.5 * IQR. (C) Comparison of dropout ratio of overlapped genes in mouse male gonadal cells in this dataset and GSE184708 dataset. (D) The number of genes detected (top) in single germ cell, and the number relative to the somatic cells of the same time‐point (bottom) were shown across the time‐points. (E) Histograms showing the relative expression levels of housekeeping gene. (F) Line plots showing the relative expression levels of well‐known germ cell markers in each cell cluster. (G) Dynamics of developmental process‐related gene sets.
**Figure S2.** related to Figure 2 (A) Violin plots showing the relative expression levels (log(TPM/10 + 1)) of *Etv5* across mitotic PGCs to Diff.ing SPG. (B) Violin plots showing the relative expression levels (log(TPM/10 + 1)) of *Usf1* across mitotic PGCs to Diff.ing SPG. (C) Immunofluorescence of ETV5 (red) co‐stained with DDX4 (green) and Hoechst (blue) in mouse male gonads. Scale bar, 100 μm. (D) Left: The quantification of relative fluorescence intensity of ETV5 related to (C). Right: the ETV5 regulon activity changing patterns from SCENIC result. (E) Immunofluorescence of USF1 (green) co‐stained with DDX4 (red) and Hoechst (blue) in mouse male gonads. Solid yellow arrowheads indicate USF1^Positive^ cells. Scale bar, 10 μm. (F) Top: The quantification of relative fluorescence intensity of USF1 related to (E). Bottom: the USF1 regulon activity changing patterns from SCENIC result.
**Figure S3.** related to Figure 3 (A) Dynamic expression of metabolism related genes represented by a self‐organizing map algorithm; divergent expression patterns of metabolism related genes are emerged in each cell cluster. A gradient of blue to red indicates low to high normalized expression value. (B) Enriched GO terms of the PGC‐related, PGC to SPG transition‐related and SPG‐related metabolism genes and P values (‐LogP).
**Figure S4.** related to Figure 4 (A) Scales probabilities and numbers of signalling pathways from germ cells to somatic cells across the time‐points. (B) The detailed enrichment of cell–cell communications at E14.5, E17.5, P2, and P7.
**Figure S5.** related to Figure 6 (A) Module dendrogram showing the different co‐expression modules. (B) Feature plots of co‐expression genes in module 1 to 12.
**Figure S6.** related to Figure 7 (A)Quantitative analysis of *Tagln2* mRNA 24 hours after transfection with non‐targeting shRNA (sh‐NC) and shRNAs against *Tagln2* in mSSCs. Data are presented as the mean ± SEM, *n* = 4, **** *p* < 0.0001, sh‐NC vs. sh‐*Tagln2*‐1 (*p* = 2.8E‐05), sh‐NC vs. sh‐*Tagln2*‐2 (*p* = 2.7E‐05), unpaired two‐tailed t test. (B) For the cell counting assay, mSSCs were initially plated at 2.5 × 10^4^ cells per well in 12‐well plates, and the total cell numbers in each well were counted from passage 1 to passage 3. Quantitative data are shown as the mean ± SEM to the right, n = at least 3, ** *p* < 0.01, *** *p* < 0.001, **** *p* < 0.0001, unpaired two‐tailed t test. (C) Flow cytometry analysis of the cell proliferation in mSSCs upon *Tagln2* knockdown using EdU proliferation kit. Quantitative data are shown as the mean ± SEM to the right, *n* = 3, **** *p* < 0.0001, unpaired two‐tailed t test. (D) Principle component analysis of RNA‐seq data from sh‐NC and sh‐*Tagln2*‐1 mSSCs. Biological replicates are highlighted.

**Table S1.** Schematic representation of the developmental time‐lines nomenclatures of germ cell in mouse and human. Modified from available literatures: PMID: 23843236 and PMID: 32950921. M, mitotic; T1, transitional 1; T2, transitional 2.

**Data S1.** Developmental process‐related gene sets.

**Data S2.** Regulon activities and clustering results.

**Data S3.** Genes related to metabolism used in SOM plot.

**Data S4.** Cell–cell interaction analysis results.

**Data S5.** NichNet analysis results.

**Data S6.** Module assignment of co‐expression analysis.

**Data S7.** GO terms of sh‐*Tagln2* and sh‐NC mSSCs.

**Data S8.** Predicted TAGLN2 regulon.

**Data S9.** Materials and oligonucleotides used.

## Data Availability

The data that supports the findings of this study are available in the supplementary material of this article.
