# Supplementary material for: Dynamic transcriptomic and regulatory networks underpinning the transition from fetal primordial germ cells to spermatogonia in mice
Source: Cell Prolif. 2024 Sep 27;58(2):e13755. doi: 10.1111/cpr.13755 (PMC11839193; doi:10.1111/cpr.13755)

Fig. S1 related to Fig. 1

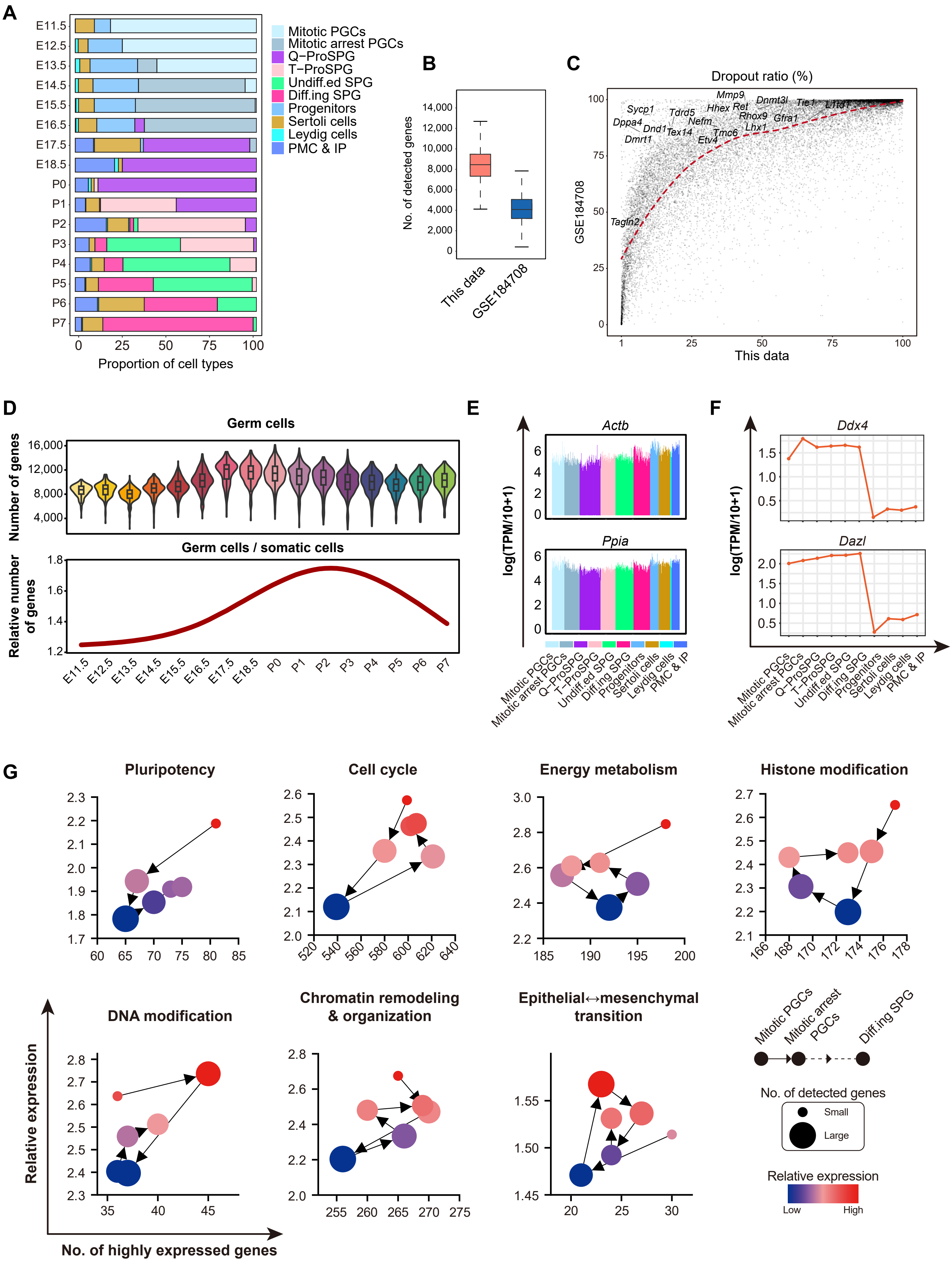

Fig. S2 related to Fig. 2

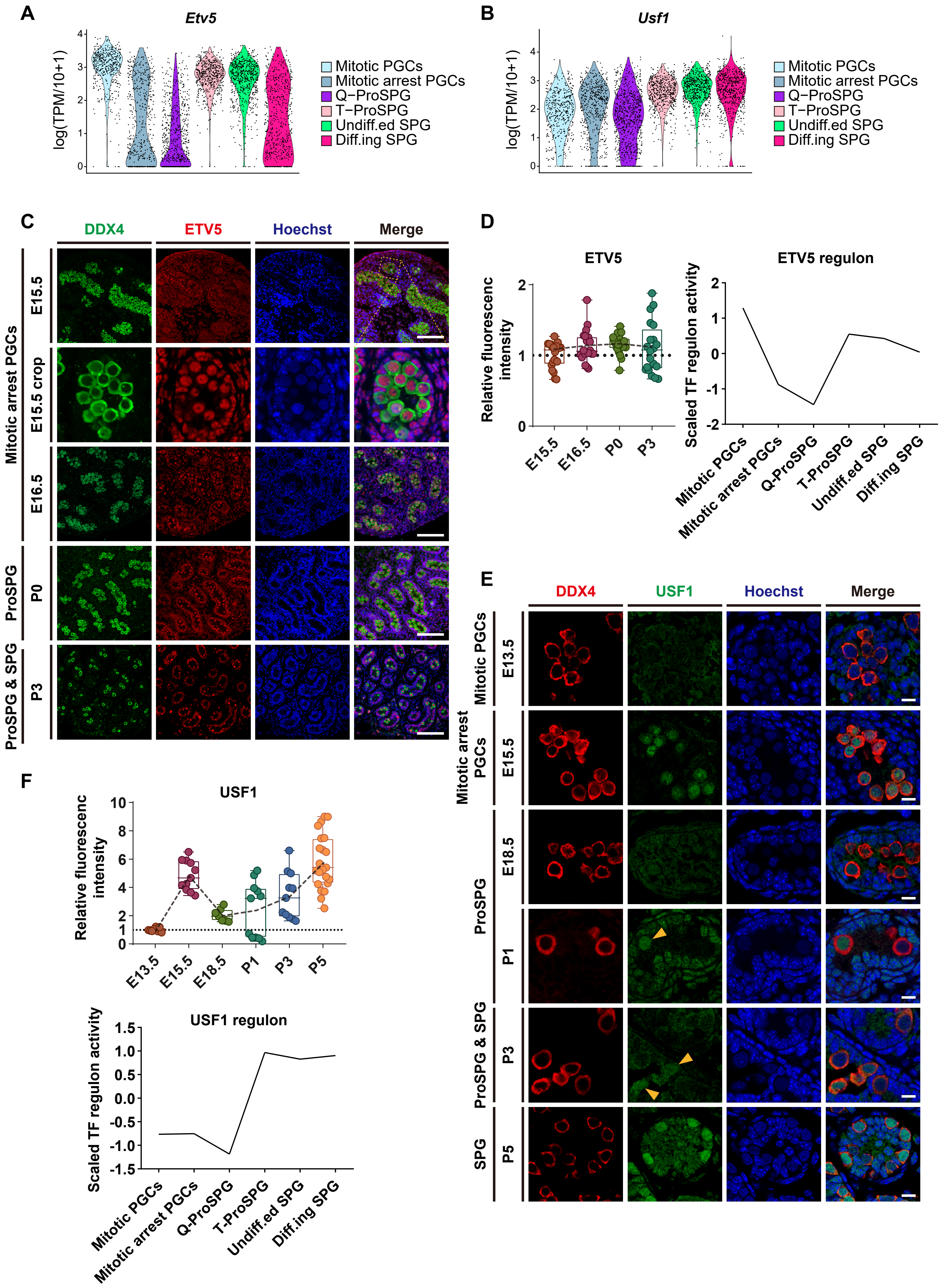

Fig. S3 related to Fig. 3

A

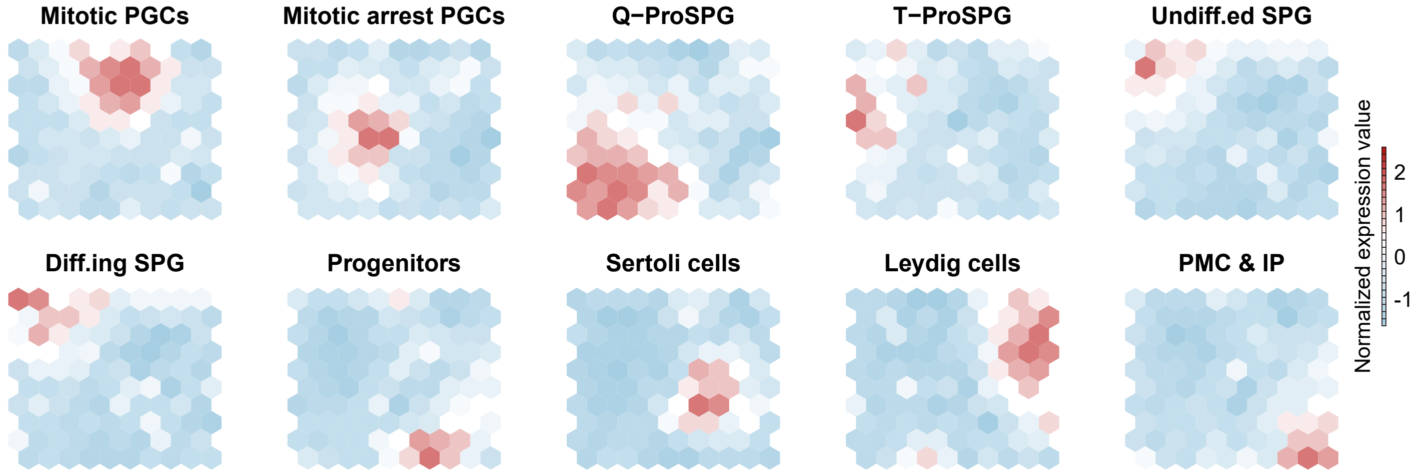

B

PGC mitosis-related

Enriched GO terms (Metascape)	-LogP
Cell Cycle	45
Organophosphate biosynthetic process	34
Regulation of chromosome organization	20
DNA Replication	17
Cellular responses to stress	11

PGC to SPG transition-related

Enriched GO terms (Metascape)	-LogP
Monoatomic ion transmembrane transport	100
Transport of small molecules	99
Monocarboxylic acid metabolic process	96
Metabolism of lipids	73
Biological oxidations	46

SPG-related

Enriched GO terms (Metascape)	-LogP
Purine metabolism	34
Cell Cycle, Mitotic	30
Amino acid metabolic process	11
Glutathione and one carbon metabolism	10
L-serine biosynthetic process	7

Fig. S4 related to Fig. 4

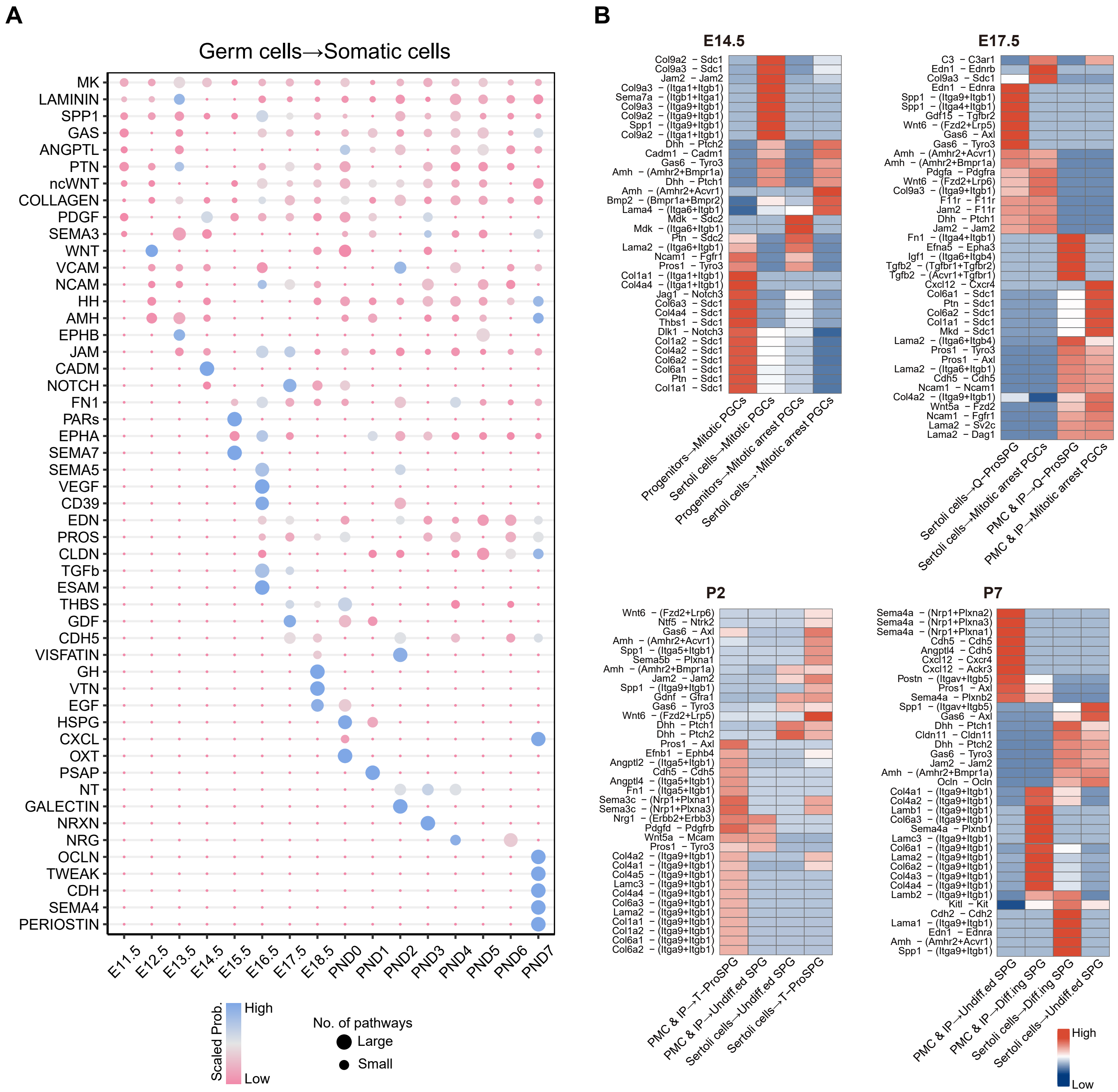

Fig. S5 related to Fig. 6

A

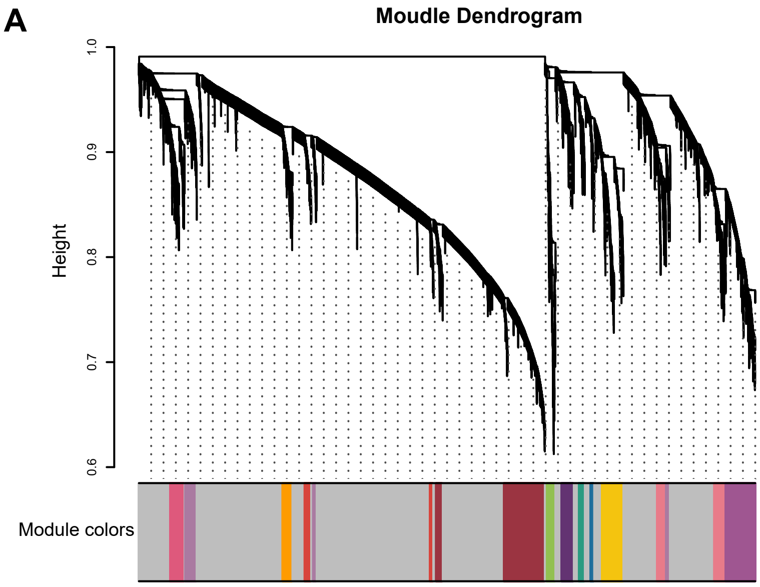

B

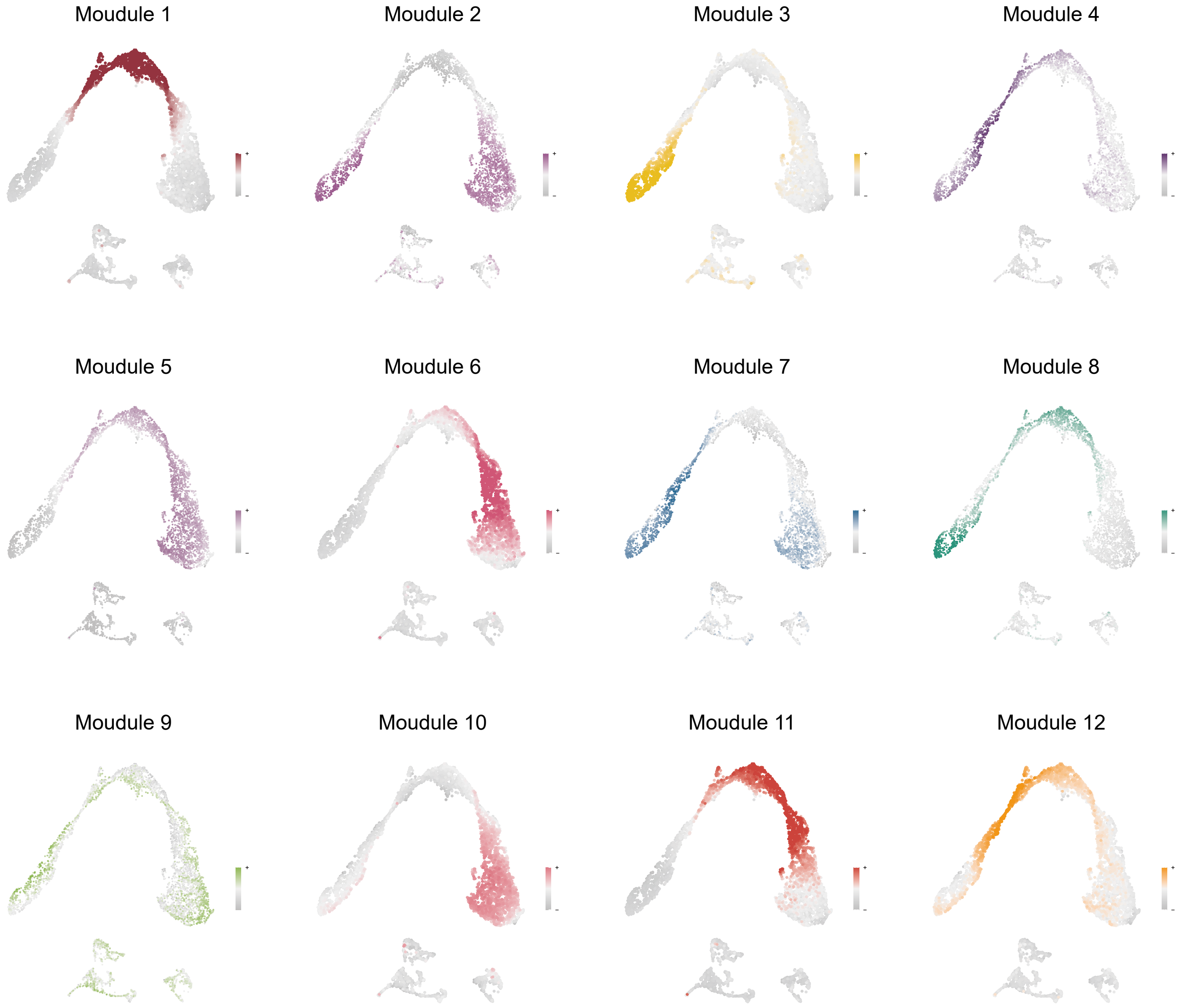

Fig. S6 related to Fig. 7

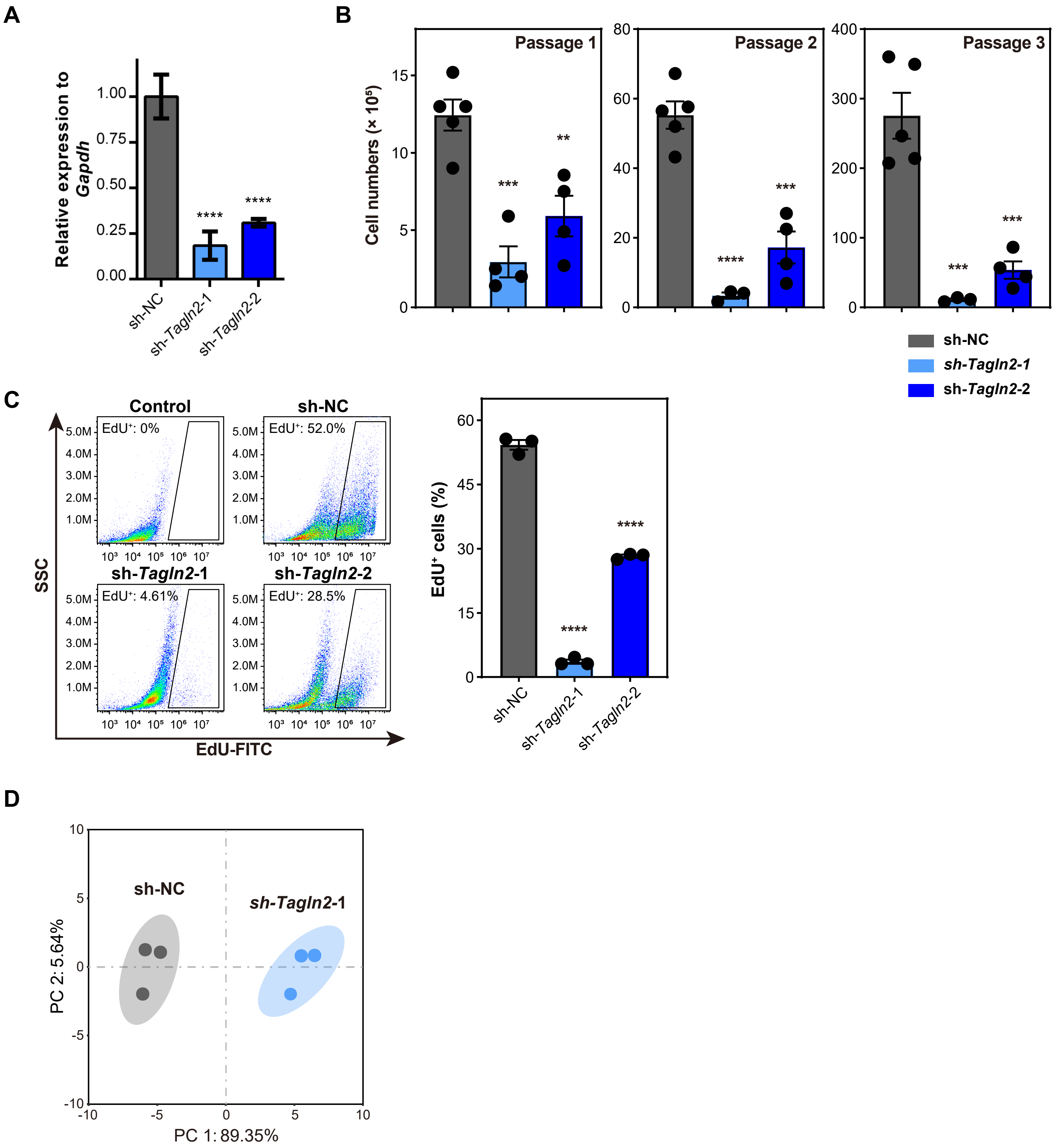

Supplement: Supplementary file 1 — Figure S1. related to Figure 1: (A) Proportion of germ cell types across the time‐points. (B) Boxplot showing the number of detected genes per cell in this dataset and GSE184708. Centre line, median; box limits, interquartile range (IQR); whiskers, minima and maxima within 1.5 * IQR. (C) Comparison of dropout ratio of overlapped genes in mouse male gonadal cells in this dataset and GSE184708 dataset. (D) The number of genes detected (top) in single germ cell, and the number relative to the somatic cells of the same time‐point (bottom) were shown across the time‐points. (E) Histograms showing the relative expression levels of housekeeping gene. (F) Line plots showing the relative expression levels of well‐known germ cell markers in each cell cluster. (G) Dynamics of developmental process‐related gene sets. Figure S2. related to Figure 2 (A) Violin plots showing the relative expression levels (log(TPM/10 + 1)) of Etv5 across mitotic PGCs to Diff.ing SPG. (B) Violin plots showing the relative expression levels (log(TPM/10 + 1)) of Usf1 across mitotic PGCs to Diff.ing SPG. (C) Immunofluorescence of ETV5 (red) co‐stained with DDX4 (green) and Hoechst (blue) in mouse male gonads. Scale bar, 100 μm. (D) Left: The quantification of relative fluorescence intensity of ETV5 related to (C). Right: the ETV5 regulon activity changing patterns from SCENIC result. (E) Immunofluorescence of USF1 (green) co‐stained with DDX4 (red) and Hoechst (blue) in mouse male gonads. Solid yellow arrowheads indicate USF1Positive cells. Scale bar, 10 μm. (F) Top: The quantification of relative fluorescence intensity of USF1 related to (E). Bottom: the USF1 regulon activity changing patterns from SCENIC result. Figure S3. related to Figure 3 (A) Dynamic expression of metabolism related genes represented by a self‐organizing map algorithm; divergent expression patterns of metabolism related genes are emerged in each cell cluster. A gradient of blue to red indicates low to high normalized expression [file CPR-58-e13755-s004.pdf]
