# Supplementary figures and images for: Dynamic transcriptomic and regulatory networks underpinning the transition from fetal primordial germ cells to spermatogonia in mice
Source: Cell Prolif. 2024 Sep 27;58(2):e13755. doi: 10.1111/cpr.13755 (PMC11839193; doi:10.1111/cpr.13755)

Table 1

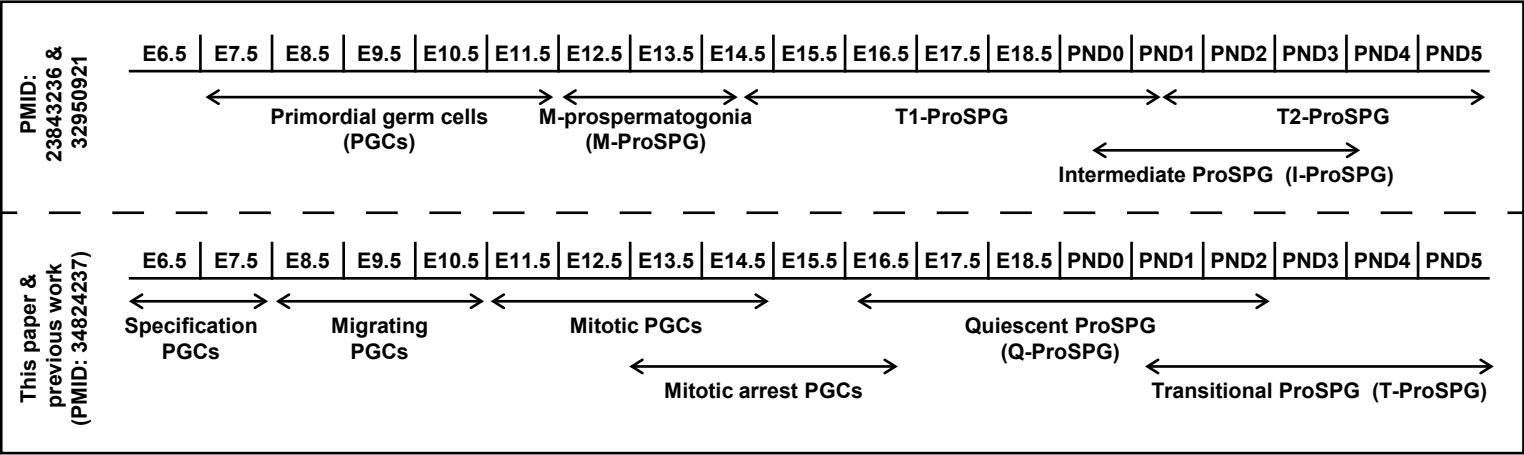

Supplement: Supplementary file 2 — Table S1. Schematic representation of the developmental time‐lines nomenclatures of germ cell in mouse and human. Modified from available literatures: PMID: 23843236 and PMID: 32950921. M, mitotic; T1, transitional 1; T2, transitional 2. [file CPR-58-e13755-s005.pdf]
